# Supplementary figures and images for: A new method of gall mite management: application of artificial defoliation to control Aceria pallida
Source: PeerJ. 2019 Mar 4;7:e6503. doi: 10.7717/peerj.6503 (PMC6404653; doi:10.7717/peerj.6503)

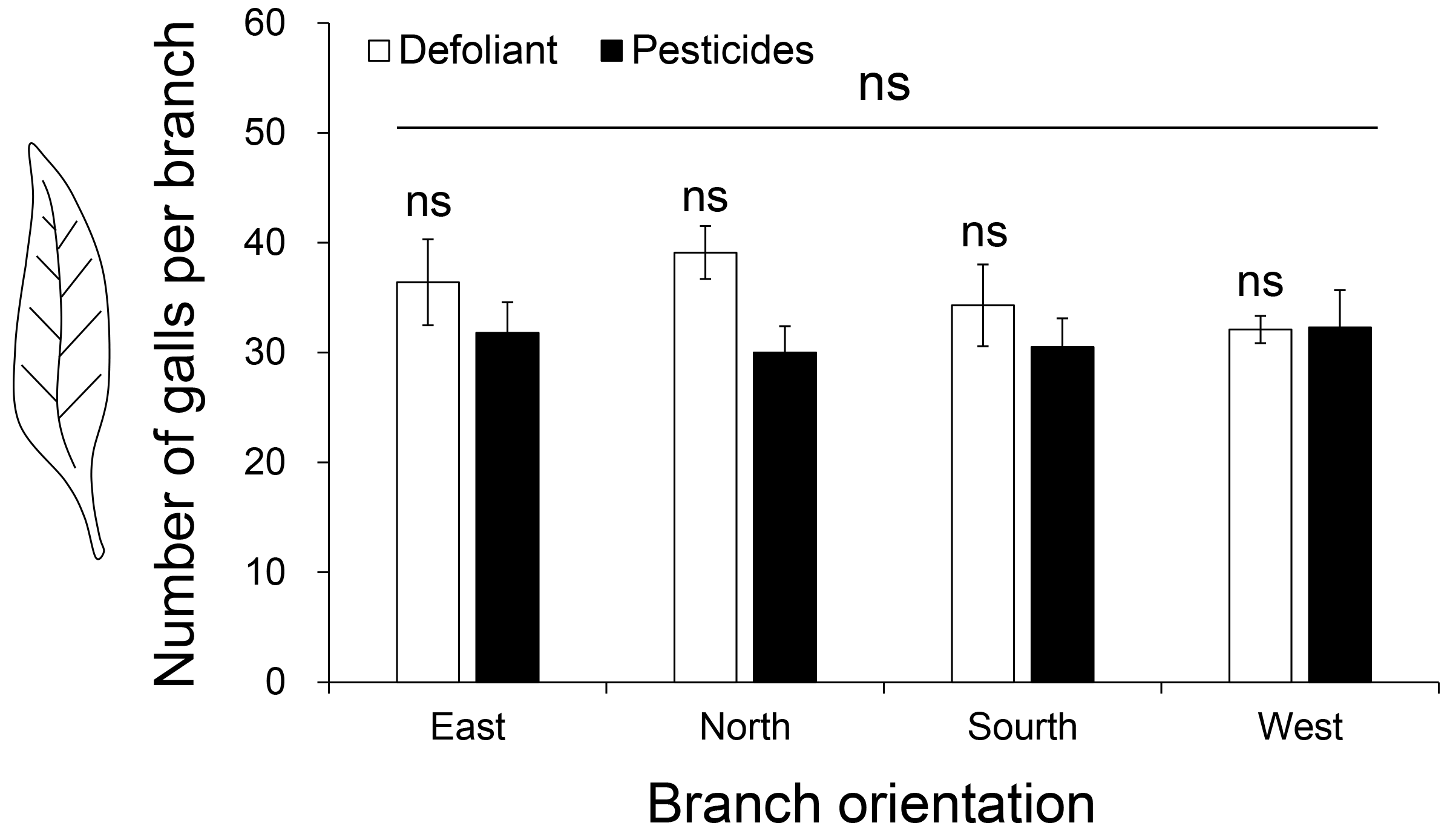

Supplement: Figure S1 — Error bars are ±SD. ns indicates no significant differences, i.e., P > 0.05. [file peerj-07-6503-s001.png]
